# Inflammatory Bowel Disease and Risk of Osteoporotic Fractures: A Meta-Analysis

**DOI:** 10.7759/cureus.5810

**Published:** 2019-09-30

**Authors:** Diego F Hidalgo, Boonphiphop Boonpheng, Jennifer Phemister, Jessica Hidalgo, Mark Young

**Affiliations:** 1 Geriatrics, Jackson Memorial Hospital / University of Miami, Miami, USA; 2 Internal Medicine, East Tennessee State University, Johnson City, USA; 3 Gastroenterology, East Tennessee State University, Johnson City, USA; 4 Internal Medicine, San Francisco De Quito University, Quito, ECU

**Keywords:** inflammatory bowel disease (ibd), osteoporosis, bone mineral density, malabsorption, osteoporotic fractures, ulcerative colitis, crohns disease

## Abstract

Introduction

Inflammatory bowel disease (IBD) and its complications have been well-established. The literature shows an association between IBD and decreased bone mineral density in the adult population. However, most studies have reported an association between IBD and osteoporosis, while the risk of fractures has not been well-studied. The aim of this meta-analysis is to summarize the best available evidence regarding IBS and osteoporotic fractures.

Methods

A review of the literature using the MEDLINE and EMBASE databases was performed during November 2017. We included cross-sectional and cohort studies that reported the relative risks, odds ratios, and hazard ratios comparing the risk of developing osteoporotic fractures among patients with IBD patients, both ulcerative colitis (UC) and Crohn’s disease (CD), versus patients without IBD as controls. The pooled odds ratio (OR) and 95% confidence interval (CI) were calculated using the generic inverse-variance method.

Results

After a review of the literature, seven studies fulfilled the eligibility criteria established during the analysis. A significant association was found between IBD and osteoporosis, with a pooled OR of 1.32 (95% CI, 1.2 - 1.4). Low heterogeneity among the studies was found, I^2^=42.3. No publication bias was found using the Egger regression test p=0.18. Sensitivity analysis showed that the inclusion of data on children by Kappelman et al. (2007) did not change the results.

Conclusion

A significant association between IBD and the risk of developing osteoporotic fractures was observed in this study. There is a 32% increased risk, which is consistent with different cohort studies previously done.

## Introduction

Inflammatory bowel disease (IBD), including ulcerative colitis (UC) and Crohn’s disease (CD), plays an important role as risk factors for the development of osteoporosis and osteoporotic fractures [1]. The risk of developing osteoporosis ranges between 17% to 41% [2]. The prevalence of both spinal trabecular and peripheral cortical osteoporosis is increased in IBD patients. In addition, there is an increased severity of clinical osteoporosis in young, amenorrheic women [3].

With the increased risk of developing osteoporosis, patients are at a greater risk of suffering osteoporotic fractures. IBD patients can have an increased risk of fractures by 40% as compared to the general population, which affects their quality of life and increases their morbidity [4-5].

The mechanism of how IBD puts patients at an increased risk of developing osteoporosis and osteoporotic fractures is likely multifactorial. Often, steroids are prescribed to patients with IBD as treatment, and this can itself predispose patients to develop osteoporosis, however, the different studies that have been adjusted for glucocorticoid use have not reported a significant increase of osteoporosis in patients taking this medication [5]. This being said, it appears to be mechanisms that intrinsically affect bone metabolic activity in patients with IBD such as the chronic inflammatory state in IBD leading to bone loss through Tumor necrosis factor (TNF) alpha RANK/RANKL/osteoprotegerin system activation, which promotes osteoclastogenesis [5]. Another contributing factor often present is secondary hyperparathyroidism due to the malabsorption of important nutrients like calcium and vitamin D [6].

The aim of this study is to summarize the best evidence correlating IBD patients with the risk of developing osteoporotic fractures versus control patients without IBD.

## Materials and methods

Search strategy

Two investigators (DH and BB) personally reviewed two databases, MEDLINE and EMBASE, during the month of November 2017. The search strategy included terms and synonyms for “IBD,” “fractures,” and “osteoporosis.”

Apart from the two major databases used for this meta-analysis, an additional manual review of the references of selected, included articles was also performed. This study meets the criteria checklist in accordance with the preferred reporting items for systematic reviews and meta-analysis (PRISMA) statement.

Selection criteria

Any study, in order to be selected for this meta-analysis, had to fulfill the following parameters:

- Case-control, cross-sectional, or cohort studies published as original articles in the two major databases used. These studies should investigate the risk of developing osteoporotic fractures in patients with IBD, either CD or UC.

- Odds ratio (OR), relative risk (RR), or hazard ratio (HR) with 95% confidence intervals (CIs) or documentation and data enough to calculate the previous ratios were provided.

**Figure 1:**
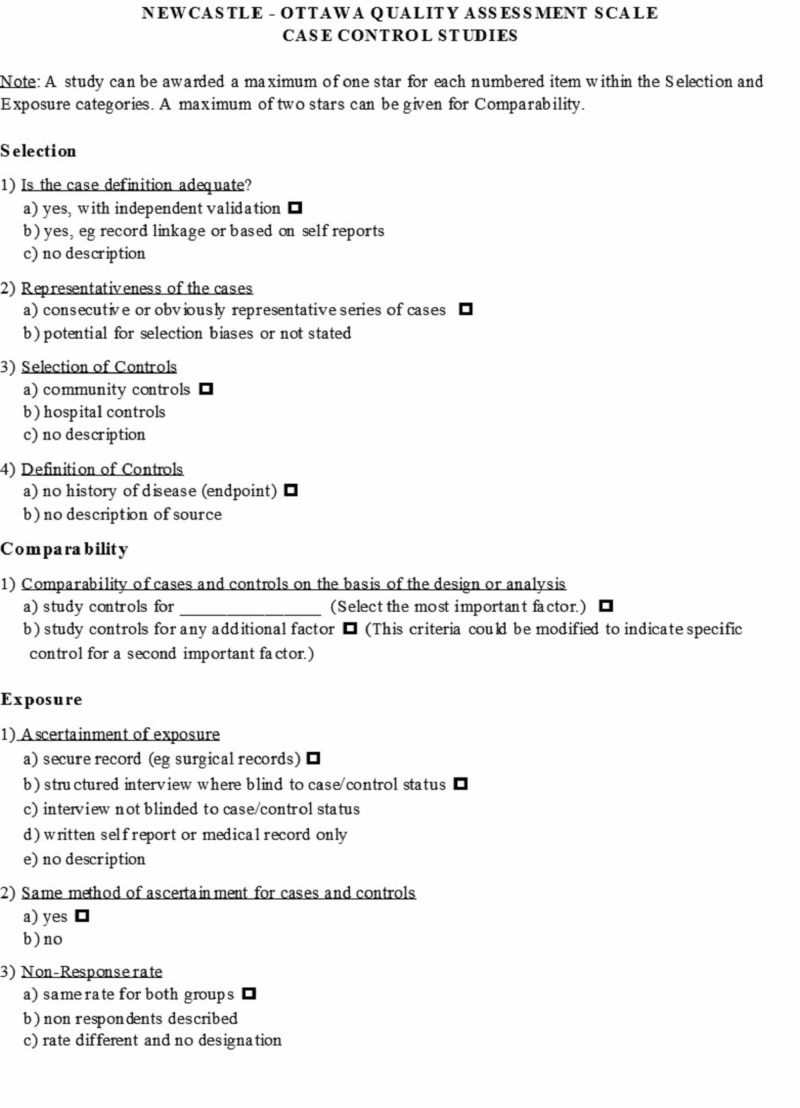
Newcastle-Ottawa quality assessment scale Adapted from "The Ottawa Hospital, Research Center," by Wells G, Shea B. 2014, http://www.ohri.ca/programs/clinical_epidemiology/oxford.asp

In order to evaluate the quality of each study, the investigators independently used the validated Newcastle-Ottawa quality assessment scale (Figure 1). This scale evaluated each study in terms of the selection of the participants, comparability between groups, as well as the ascertainment of the exposure of interest for case-control studies, and the outcome of interest for cohort studies [7].

Data extraction

A Microsoft Excel (Microsoft Corporation, Redmond, Washington, US) data collection form was used to summarize the most relevant information obtained from these studies. This table contained the first author's last name, the country where the study was conducted, year of publication, demographics, the Newcastle-Ottawa quality assessment scale, the total number of participants, characteristics of the participants, the method used to diagnose IBD, the method used to determine fractures, adjusted effect estimates with 95% CI, and covariates that were adjusted in the multivariate analysis (Table 1).

**Table 1:** Characteristics of studies included in the analysis. Abbreviation: IBD: Inflammatory Bowel Disease, UC: Ulcerative Colitis, CD: Crohn’s Disease, DXA: Dual X-Ray Absorptiometry, MBD: Metabolic Bone Disease, BMD: Bone Metabolic Density, UMIBDED: University of Manitoba IBD Epidemiologic Database

	Bernstein et al. 2000 [5]	Edwards et al. 2002 [8]	Kappelman et al. [9]	Targownik et al. 2013 [10]	Tjeerd-Pieter et al. 2003 [11]	Ferencz et al. 2002 [12]	Tsai et al. 2014 [13]
Country	Canada	USA	USA	Canada	United Kingdom	Hungary	China
Study design	Population-based cohort study	Population-based inception cohort study	Cross-sectional	Population-based cohort study	Primary care-based nested case-control study	Population-based cohort study	Population-based cohort
Year	2000	2002	2010	2013	2003	2002	2014
Number of participants	6027	238	1242	1230	231,778	110	3141 IBD patients and 12,564 age- and sex-matched controls
Participants	Patients with inflammatory bowel disease in the University of Manitoba IBD Database (n 5 6027) were matched to 10 randomly selected persons in the general population without inflammatory bowel disease (n 5 60 270) by year, age, sex, and postal area of residence.	238 patients with a diagnosis of Crohn’s Disease based on clinical records.	733 children with CD, 488 with UC, and 3287 controls.	we included only the first DXA examination that any patient underwent between 1997 and 2008. Subjects in the MBMDD who were also cases in the UMIBDED and were diagnosed with IBD before the date of the DXA examination were considered to have IBD, whereas all other subjects were considered to not have IBD. Subjects with IBD were also further sub-classified as having CD or UC.	Case patients were permanently registered Patients aged 18 years and older who had a fracture at any site in their medical records. Control patients were adults without a history of fracture in their medical records.	110 IBD patients, 80 ( UC) and 30 (Crohn’s disease) were involved in the study. 110 patients with suspected MBD due to other reasons and regularly referred for BMD measurement were used for comparison.	Patients aged 20 years and older with IBDs, namely UC (ICD-9-CM code 556) and CD (ICD-9-CM codes 555.0–555.2), who were newly diagnosed between 2000 and 2010, and refer to them as the IBD cohort. For each IBD patient, four comparisons were randomly selected from the pool of participants without IBD and osteoporosis.
Mean age of participants in years	39	33.4 +/-16.3 years (range, 4–84 years).	15	61	68.4	35.2±17 years.	47
Percentage of female	NA	54%	45%	76%	28%	50%	47%.
Diagnosis of IBD	Clinical records from Manitoba IBD database obtained from Manitoba Health Administrative Database. ICD 9 CM codes were used to identify patients with either Crohn’s disease or ulcerative colitis.	Complete medical records of each candidate case were retrieved and reviewed for the diagnosis of CD between 1940 and 1993 from Olmsted County residents.	Patients with at least three health care contacts, on different days, associated with ICD codes for IBD. Or patients with at least one claim for CD or UC and at least one pharmacy claim for any of the following medications: mesalamine, olsalazine, balsalazide, sulfasalazine, 6-mercaptopurine, azathioprine, infliximab, adalimumab, or enteral budesonide.	Medical Records from The University of Manitoba IBD Epidemiologic Database (UMIBDED) that mentioned the ICD codes for IBD, Crohn’s and ulcerative colitis.	Severity of IBD was assessed using 2 factors: a history of general practitioner visits for symptoms of IBD (diarrhea, abdominal pain, anemia, rectal bleeding, or weight loss) and hospitalization for a gastrointestinal disorder in the 12 months before the index date. In addition, the use of medication for the treatment of IBD was examined	NA	Records review of 20 years and older patients with IBDs, namely UC (ICD-9-CM code 556) and CD (ICD-9-CM codes 555.0–555.2), who were newly diagnosed between 2000 and 2010, and refer to them as the IBD cohort.
Diagnosis of Fractures/Osteoporosis	Using the Manitoba Health administrative database for hospital discharge abstracts (using ICD-9-CM codes for hip fracture, also outpatient visits for rib, spine and wrist/forearm codes.	Radiologist’s reports about Fractures of the hip, spine, or distal forearm that resulted from minimal or moderate trauma in patients 35 years or older were considered to represent osteoporotic fractures.	Claims for fractures occurring at each of the following sites using ICD-9 diagnosis codes: ankle, clavicle, foot, hand, humerus, femur, radius, skull, tibia, and vertebral.	A subject is considered to have DXA-defined osteoporosis if the T score is less than _2.5.	Registered patients aged 18 years and older who had a fracture at any site in their medical records.	BMD of the lumbar spine, hip, and proximal forearm were measured by Prodigy (GE Lunar) densitometer, and Z-scores were evaluated. Previous low- trauma fractures were also registered.	The IBD cohort and the non-IBD cohort were followed up until osteoporosis was diagnosed or censored because of mortality, loss to follow-up, withdrawal from the insurance system, or December 31, 2010. Osteoporosis was diagnosed according to a T score with >-2.5 SD.
Adjusted OR or HR or IRR	(IRR, 1.41 (CI, 1.27 to 1.56); P < 0.001).	risk ratio for any fracture was 0.9 (95% confidence interval (CI), 0.6– 1.4),	CD odds ratio (OR) 0.8, 95% confidence interval (CI)0.6–1.1; UC OR 1.4, 95% CI 1.0–2.1)	(OR), 1.20; 95% confidence interval (CI), 1.02–1.40).	(odds ratio (OR), 1.72; 95% confidence interval (CI), 1.13–2.61)	(RR: 1.41, CI 1.27–1.56). P <0.001	(Adjusted hazard ratio (AHR), 1.31; 95% CI, 1.09–1.60, p = 0.004).
Confounder adjustment	Controls were matched to persons with inflammatory bowel disease by year of birth, sex, and postal area of residence at the date of diagnosis of the index case of inflammatory bowel disease.	Sex, age, cigarette smoking, body weight, height, use of corticosteroids, small bowel involvement, colonic involvement, ileocolonic involvement)	Age, gender, and geographical region	Age, sex, body mass index, hormone replacement therapy, osteoprotective medications, and corticosteroid use.	age (within 1 year) and sex	Sex, age	Age- and sex-matched controls
Quality assessment (Newcastle-Ottawa scale)	Selection: 4 Comparability:1 Outcome: 4	Selection: 4 Comparability: 1 Outcome: 3	Selection: 3 Comparability: 1 Outcome: 2	Selection: 4 Comparability: 1 Outcome: 2	Selection: 3 Comparability: 1 Outcome: 3	Selection: 3 Comparability: 1 Outcome: 2	Selection: 4 Comparability: 1 Outcome: 3

To ensure accuracy, all investigators performed the data extraction process independently. Any data discrepancy was also resolved by referring back to the original articles.

Statistical analysis

Data analysis was performed using Review Manager 5.3 software from the Cochrane Collaboration (London, United Kingdom). Adjusted point estimates and standard errors from the individual study were combined using the generic inverse variance method of DerSimonian and Laird, which assigned the weight of each study based on its variance [14]. In light of the possible high between-study variance due to different study designs and populations, we used a random-effect model rather than a fixed-effect model. Cochran's Q test and I2 statistic were used to determine the between-study heterogeneity. A value of I2 of 0%-25% represents insignificant heterogeneity, greater than 25% but less than or equal to 50%, represents low heterogeneity, greater than 50% but less than or equal to 75% represents moderate heterogeneity, and greater than 75% represents high heterogeneity [15].

## Results

**Figure 2:**
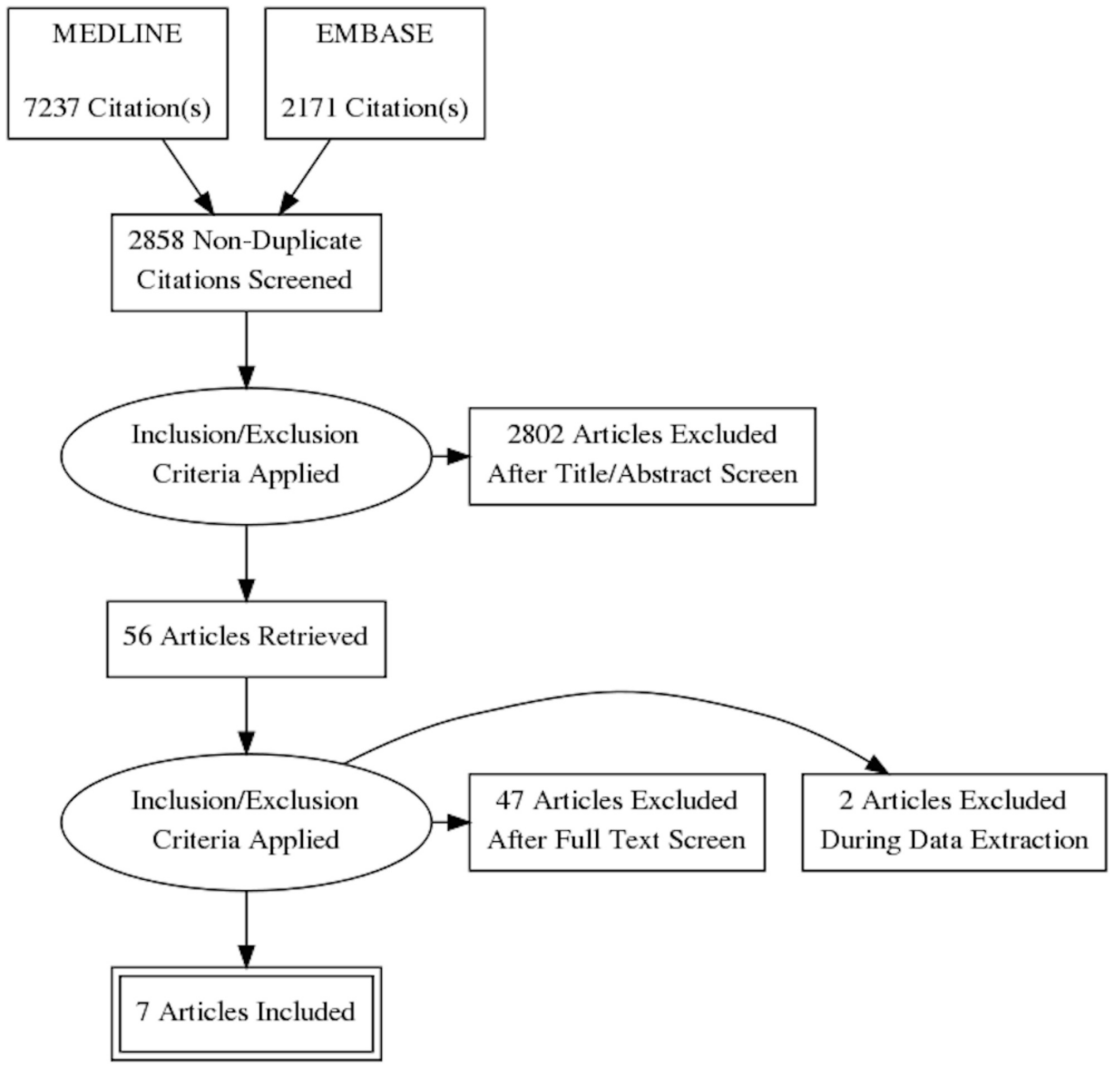
Search criteria and eligibility

An advanced search yielded 7237 articles on Medline and 2171 articles on EMBASE. After the exclusion of 6550 articles that were duplicated, 2858 underwent a title and abstract review. A total of 2802 articles were excluded, as they were case reports, book articles, letters to the editor, or review articles without the information needed for the analysis, leaving 56 articles for a full-length article review. A total of 45 articles were dismissed at this time, as they did not report the outcome we were looking for. Two articles did not have comparators so they were also excluded. A total of seven studies were used for statistical analysis [16], five of them were cohort studies, one case-control study, and one cross-sectional study. The outlines of the literature review and study selection process is outlined in Figure 2. The clinical characteristics of each study and the quality assessment are described in Table 1.

**Figure 3:**
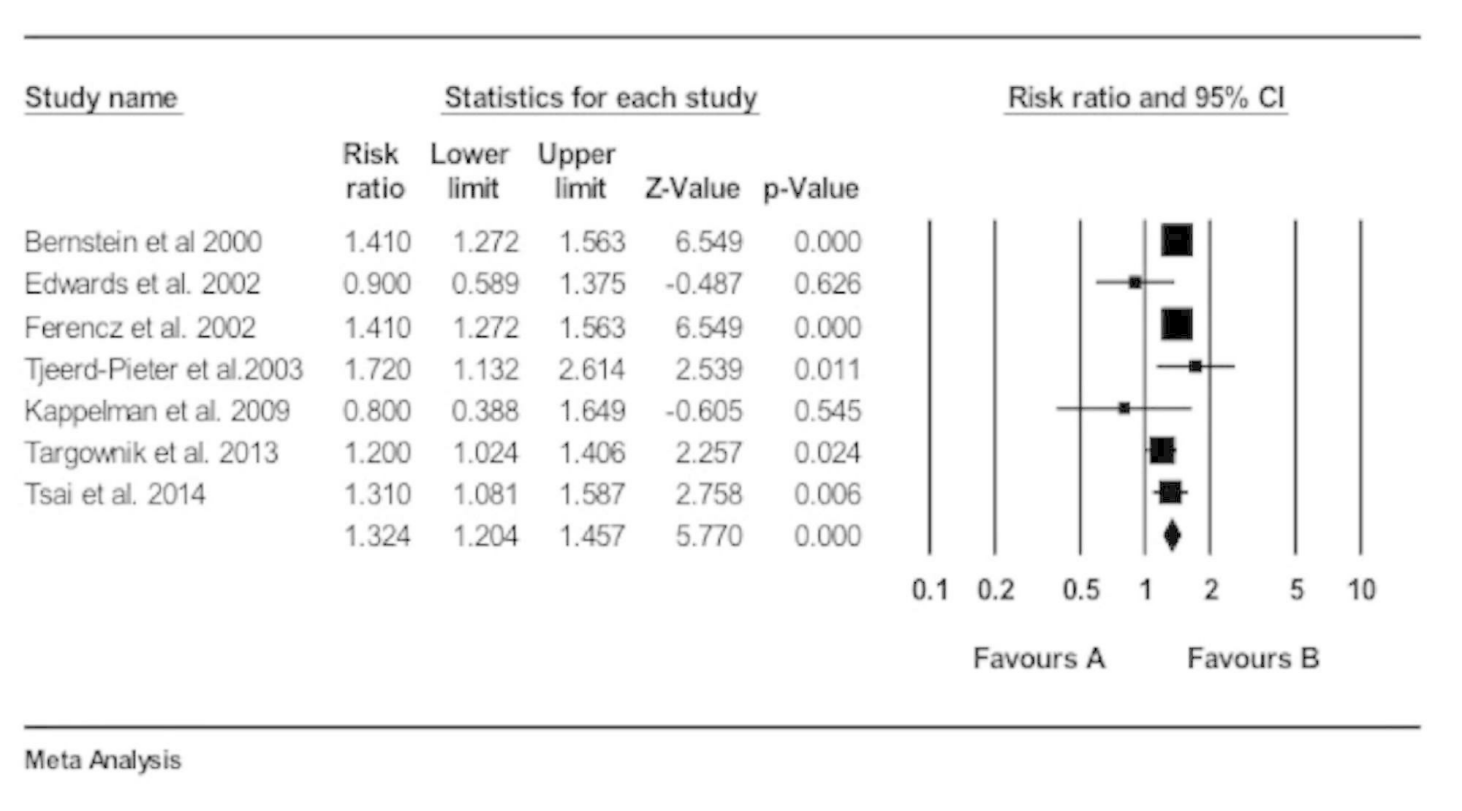
Relative risk and P values

The overall analysis found a higher risk of osteoporotic fractures in patients with IBD, either UC or CD, as compared with the control individuals who did not have IBD. The odds ratio (OR) was 1.32 (95% CI: 1.20 - 1.45), P <0.001, as shown in Figure 2. Sensitivity analysis showed that the inclusion of data on children by Kappelman et al. (2009) did not affect the results, as shown in Figure 3.

To measure heterogeneity among the studies, Cochran’s Q test and I2 were calculated. The I2 calculated for this study was 42.3, which represents low heterogeneity among the studies.

**Figure 4:**
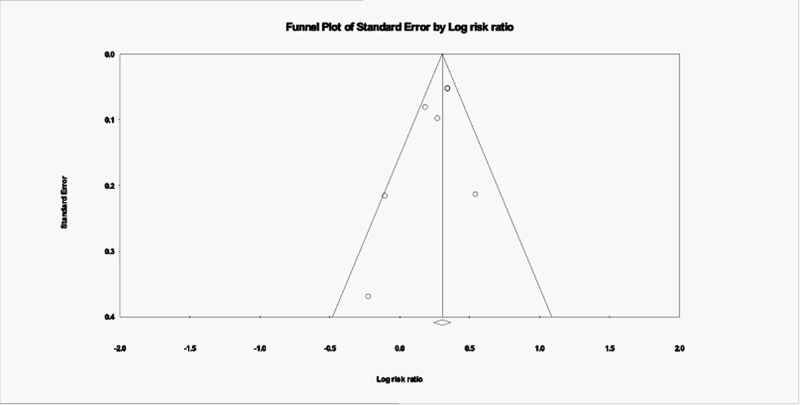
Publication bias; Funnel plot

The Egger regression test and funnel plots were used to assess publication bias [17]. Egger's regression test (P 0.18) did not show a publication bias. Funnel plots (Figure 4) were symmetrical, indicating low publication bias. The total number of studies were seven, which correlates with high power for this test.

## Discussion

This study is a meta-analysis to evaluate the risk of developing osteoporotic fractures among IBD patients. After evaluating a total of seven studies (cohort, case-control, and cross-sectional) that met the inclusion criteria, with a total population of 242,524 patients, we found that patients with IBD have a 32% increased risk of developing osteoporotic fractures.

This is the first meta-analysis that assesses the relationship between IBD and fractures. A lot of data has been obtained to assess the relationship between IBD and osteoporosis, which is defined as reduced mineral density. However, the risk for developing bone fractures is the one associated outcome that really has significant morbidity, mortality, and healthcare cost [16,18].

This meta-analysis takes into consideration research studies done all over the world, including the USA, UK, Hungary, China, and others. It collects more than 200,000 patients and records. We included a study done by Kappelman et al. (2009) that took into consideration the pediatric population [9]. This study showed no increased risk of fractures in patients below the age of 20. Sensitivity analysis showed that the inclusion of this article in the pool of information did not have a big impact on the final outcome. These results confirm that the risk of osteoporotic fractures increases with age, and even patients with IBD tend not to show accelerated signs of bone disease until years after the disease has progressed [19].

We calculated the risk of fractures on IBD as a whole. We did not make any distinction between CD or UC, as some articles were just focused on CD while others included UC too. Further studies would be needed to evaluate the risk of fractures associated with the specific type of IBD.

The data used for this meta-analysis were matched for the use of corticosteroids. Steroids by themselves put patients at increased risk of fractures. The most significant effect of steroids n the bones is inhibition of osteoblastic activity, which decreases bone formation. This inhibition in the number of osteoblasts is secondary not only to a decrease in the formation but also an increase in the death of mature osteoblasts. At the same time, glucocorticoids decrease the function of the remaining osteoblasts directly and indirectly through the inhibition of insulin-like growth factor I expression [20]. Since most IBD patients are or have been on steroids at some point of the disease, this could be a confounding factor. Direct standardization was used to adjust for this confounder.

After adjusting for the use of this medication, the decrease in bone mass and the increase of developing bone fractures was significant enough to confirm that an inflammatory state is enough to make changes to the bone metabolism.

A low I2 value on the statistical analysis suggests that the variation across studies is due to a low heterogeneity rather than chance.

More studies should be done to calculate the risk to develop fractures in the pediatric population since early identification, risk factors modification, and treatment could slow the risk of bone mass loss and improve morbidity in that specific population.

## Conclusions

A significant association between IBD and the risk of developing osteoporotic fractures was observed in this study. There is a 32% increased risk of developing osteoporotic fractures in patients with IBD as compared to individuals without IBD used as controls. There was no significant increase of osteoporosis in IBD patients taking corticosteroids after this variable was adjusted through direct standardization.
